# Identifying Key Performance Indicators for Holistic Hospital Management with a Modified DEMATEL Approach

**DOI:** 10.3390/ijerph14080934

**Published:** 2017-08-19

**Authors:** Sheng-Li Si, Xiao-Yue You, Hu-Chen Liu, Jia Huang

**Affiliations:** 1 School of Economics and Management, Tongji University, Shanghai 200092, China; sishengli@aliyun.com (S.-L.S.); youxiaoyue@gmail.com (X.-Y.Y.); 2 Institute for Manufacturing, University of Cambridge, Cambridge CB3 0FS, UK; 3 School of Management, Shanghai University, Shanghai 200444, China; jiahuangshu@foxmail.com

**Keywords:** hospital management, key performance indicator (KPI), evidential reasoning, decision making trial and evaluation laboratory (DEMATEL)

## Abstract

Performance analysis is an important way for hospitals to achieve higher efficiency and effectiveness in providing services to their customers. The performance of the healthcare system can be measured by many indicators, but it is difficult to improve them simultaneously due to the limited resources. A feasible way is to identify the central and influential indicators to improve healthcare performance in a stepwise manner. In this paper, we propose a hybrid multiple criteria decision making (MCDM) approach to identify key performance indicators (KPIs) for holistic hospital management. First, through integrating evidential reasoning approach and interval 2-tuple linguistic variables, various assessments of performance indicators provided by healthcare experts are modeled. Then, the decision making trial and evaluation laboratory (DEMATEL) technique is adopted to build an interactive network and visualize the causal relationships between the performance indicators. Finally, an empirical case study is provided to demonstrate the proposed approach for improving the efficiency of healthcare management. The results show that “accidents/adverse events”, “nosocomial infection”, ‘‘incidents/errors”, “number of operations/procedures” are significant influential indicators. Also, the indicators of “length of stay”, “bed occupancy” and “financial measures” play important roles in performance evaluation of the healthcare organization. The proposed decision making approach could be considered as a reference for healthcare administrators to enhance the performance of their healthcare institutions.

## 1. Introduction

In the past decades, healthcare systems have been involved in many changes ranging from technological to normative. But various issues have been raised in the healthcare sector and the operation of hospital management needs great improvement. As evidence for how well organizational objectives or goals are achieved, performance measurements have emerged in healthcare organizations [1,2]. Evaluating clinical and service performance is significant to drive quality excellence and achieve more effective performance. Thus, many national indicator projects [3,4] as well as international projects [5,6] have been initiated for measuring the quality and performance of healthcare services. These existing performance assessment frameworks include a large number of indicators [7], but it is difficult to improve them simultaneously since the resources of hospital management are constrained. Furthermore, the optimization of a specific process deviates from the overall welfare of a whole system, but few of the current frameworks can analyze and enhance healthcare performance systematically. Therefore, it is of great importance to determine a limited number of essential performance indicators based on their influences and relationships to address the management issues of healthcare organizations from a holistic point of view. 

In previous studies, key performance indicators (KPIs) for healthcare management are commonly identified through case studies or interviews with healthcare experts [4,8,9]. However, these studies mainly focus on a generic framework of screening effective indicators for performance measurement; almost none has examined the creation of a mechanism that distinguishes causal relationships between KPIs. In other words, there is a lack of articulation of the cause and effect relationships between performance indicators when implemented for performance improvement. Consequently, many hospitals dilute the efforts of their performance measurement systems because of basic mistakes in mapping [10]. The decision making trial and evaluation laboratory (DEMATEL) method [11], initiated by the Geneva Research Centre of the Battelle Memorial Institute, is pragmatic to visualize the structure of complicated causal relationships and clarify the essentials of a system. It is a method based on digraph and matrix theory and has the ability to divide multiple factors into cause and effect groups [12]. Through analyzing direct and indirect relations between system elements, an ideal way to solve the intertwined problems can be obtained by the DEMATEL [13,14,15]. Although there is an abundance of literature using DEMATEL, this effective structural modeling tool has not yet been applied to the healthcare performance management. Essentially, for a large number of indicators impacting each other, healthcare performance measurement can be considered as a complex system. Hence, it is an interesting research topic for applying the DEMATEL technique to capture the network of interdependencies among performance indicators and identify KPIs.

In many real-life situations, experts’ judgments are vague and it is difficult to estimate direct effect between elements with an exact numerical value. Instead, domain experts feel more comfortable providing their knowledge with linguistic expressions due to the complexity of the considered objects. Moreover, they may demonstrate different opinions from each other and produce different types of assessment information of performance indicators, some of which may be precise or imprecise, certain or uncertain, and complete or incomplete. These various types of linguistic assessments are very difficult to incorporate into the classical DEMATEL approach. The evidential reasoning (ER) approach was developed based on Dempster-Shaffer theory and distributed assessment framework for analyzing decision making problems with different types of uncertainties [16,17]. It can handle various kinds of human judgments such as uncertainty, fuzziness and ignorance and allow decision makers to express opinions in a flexible manner. Due to its characteristics and benefits, the ER approach has been widely applied to address decision-making problems in different areas [18,19]. To manage linguistic information without loss of information, the 2-tuple linguistic computational model was proposed by Herrera, Martínez [20] for computing with words. A well-known extension of 2-tuple linguistic method is the interval 2-tuple linguistic model [21], which employs uncertain linguistic variables to represent linguistic evaluation information. Recently, many of studies have reported decision-making models under the interval 2-tuple linguistic setting [22,23]. Consequently, the normal DEMATEL method can be extended with the ER and interval 2-tuples for more accurately constructing the network structure of interdependent healthcare performance indicators. 

Based on the above analyses, this paper aims to develop a hybrid multiple criteria decision-making (MCDM) approach, called linguistic evidential DEMATEL, to analyze the total relations of performance indicators and identify KPIs for healthcare performance improvement. For the convenience of expressing uncertainties, linguistic assessments provided by domain experts are modeled via the ER approach. The interval 2-tuple linguistic model is utilized to aggregate individual opinions of experts into group assessments. Next, the DEMATEL technique is applied on the group assessment matrix, and the indicators that are of more essential importance for the whole system can be recognized. For doing so, the remainder of this paper is organized as follows: Section 1 reviews the literature on interval 2-tuples and the DEMATEL method. Section 2 introduces some basic concepts and definitions which will be utilized in this study. In Section 3, we present an extended DEMATEL approach to identify KPIs for holistic hospital management. In Section 4, a case study is conducted to illustrate the application of the proposed hybrid approach. Finally, conclusions and future research directions are given in the Section 5.

## 2. Literature Review

### 2.1. Applications of Interval 2-Tuples

Since its introduction, the interval 2-tuple linguistic method has received great attention from researchers and has been applied to many fields. For example, Xue et al. [24] combined hesitant fuzzy linguistic term sets with interval 2-tuples to deal with the hesitancy and uncertainty of decision makers and developed a QUALIFLEX-based linguistic method for robot evaluation and selection. Singh et al. [23] addressed the energy-planning problem in a new power plant set up by extending the PROMETHEE II approach to the interval 2-tuple linguistic environment. Lu et al. [25] solved the healthcare waste treatment technology selection problem with a hybrid decision-making model using interval 2-tuple induced distance operators and technique for order preference by similarity to an ideal solution (TOPSIS) method. You et al. [26] proposed an interval 2-tuple linguistic VIKOR approach for group multi-criteria supplier selection within incomplete and uncertain information environment, and Liu et al. [27] presented an attitudinal-based interval 2-tuple linguistic VIKOR method to select the best disposal site in municipal solid waste management. Liu et al. [28] suggested a failure mode and effect analysis (FMEA) method using interval 2-tuple linguistic variables and grey relational analysis (GRA), and Liu et al. [29] assessed the risk of healthcare failure modes based on interval 2-tuple hybrid weighted distance measure. In addition, Meng et al. [30] defined some generalized interval 2-tuple linguistic correlated aggregation operators based on Choquet integral and generalized Shapley function for solving the multiple criteria decision-making (MCDM) problems where the elements in a set are interdependent. Wang et al. [31] integrated interval 2-tuple linguistic method with Choquet integral aggregation operators to deal with the linguistic MCDM problems with correlated criteria. Lin et al. [32] put forward the generalized interval 2-tuple linguistic Shapley chi-square averaging operator to cope with interactive phenomenon among experts (or attributes) in MCDM. Liu et al. [33] proposed some interval 2-tuple linguistic Bonferroni mean operators for MCDM considering the interrelationships among input arguments, and Liu et al. [34] defined some dependent interval 2-tuple linguistic aggregation operators, in which the associated weights only depend on the input arguments and can relieve the effect of biased arguments on the decision results.

### 2.2. Applications of the DEMATEL Method

In the literature, a lot of researchers have adopted the decision making trial and evaluation laboratory (DEMATEL) method for the identification of key factors through analyzing the interaction relationships among them. For instance, Luthra et al. [35] evaluated the recognized key enablers in solar power initiatives and developments in India’s current scenario based on fuzzy DEMATEL methodology. Liang et al. [36] identified the critical success factors influencing the sustainable development of biofuel industry in China using grey DEMATEL approach and suggested appropriate strategic measures to promote the sustainability of biofuel industry in China. Xia et al. [37] employed grey DEMATEL method to analyze the significant internal barriers for automotive parts remanufacturers in China and help them increase the chance of practicing more productive remanufacturing. Wu et al. [38] used fuzzy DEMATEL method to investigate the decisive factors for green supply chain practices implementation in the Vietnamese automobile manufacturing industry. Liu et al. [39] determined critical failure modes in system FMEA by combining fuzzy weighted average with fuzzy DEMATEL model, and Guo et al. [40] evaluated green corporate social responsibility indicators and the degrees of influence and causal relationships among them via a novel DEMATEL technique. Akyuz, Celik [41] adopted fuzzy DEMATEL approach to evaluate critical operational hazards during gas freeing process in crude oil tankers, and Wu [42] utilized the DEMATEL and balanced scorecard (BSC) methods to identify critical central and influential KPIs for improving banking performance. Cheng et al. [43] investigated the concepts and relationships of KPIs through mind map and extract KPIs of new product development by analyzing their cause and effect relationships. Additionally, Govindan et al. [44] developed an intuitionistic fuzzy DEMATEL method for handling the important and causal relationships between green supply chain management practices and performances. Quader et al. [45] used a 2-tuple DEMATEL technique to evaluate the top influencing factors for CO_2_ capture and storage in the iron and steel industry. Li et al. [46] developed an evidential DEMATEL method based on intuitionistic fuzzy numbers and Dempster-Shaffer theory to identify critical success factors in emergency management.

## 3. Preliminaries

### 3.1. Interval 2-Tuple Linguistic Variables

The interval 2-tuple linguistic model [21] is an extension of 2-tuple linguistic approach [20,47] for better representing uncertain linguistic decision information. The main advantages of this formalism are that decision makers can express their judgments by the use of different linguistic term sets, and various uncertainties in the assessments of decision makers can be well reflected and modeled [48,49,50].

**Definition** **1.** *Let* $S=\left\{s_{0},s_{1},...,s_{g}\right\}$ *be a linguistic term set. An interval 2-tuple linguistic variable is composed of two 2-tuples, denoted by* $\left[\left(s_{k},\alpha_{1}\right),\left(s_{l},\alpha_{2}\right)\right]$*, where* $\left(s_{k},\alpha_{1}\right)\leq \left(s_{l},\alpha_{2}\right)$*,* $s_{k}\left(s_{l}\right)$ *and* $\alpha_{1}\left(\alpha_{2}\right)$ *represent the linguistic label of the linguistic term set S and symbolic translation, respectively. The interval 2-tuple that expresses the equivalent information to an interval value* $\left[\beta_{1},\beta_{2}\right]\left(\beta_{1},\beta_{2}\in \left[0,1\right],\beta_{1}\leq \beta_{2}\right)$ *is computed by the following function [21]:*

(1) $$\Delta \left[\beta_{1},\beta_{2}\right]=\left[\left(s_{k},\alpha_{1}\right),\left(s_{l},\alpha_{2}\right)\right]\;\mathrm{with}\;\{\begin{array}{l} s_{k},\;k=\mathrm{round}\left(\beta_{1}\cdot g\right)\\ s_{l},\;l=\mathrm{round}\left(\beta_{2}\cdot g\right)\\ \alpha_{1}=\beta_{1}-\frac{k}{g},\;\alpha_{1}\in \left[-\frac{1}{2g},\frac{1}{2g}\right)\\ \alpha_{2}=\beta_{2}-\frac{l}{g},\;\alpha_{2}\in \left[-\frac{1}{2g},\frac{1}{2g}\right) \end{array}.$$

On the other hand, there is always a function $\Delta^{-1}$ so that an interval 2-tuple can be converted into an interval value $\left[\beta_{1},\beta_{2}\right]\left(\beta_{1},\beta_{2}\in \left[0,1\right],\beta_{1}\leq \beta_{2}\right)$ by the relation.

(2) $$\Delta^{-1}\left[\left(s_{k},\alpha_{1}\right),\left(s_{l},\alpha_{2}\right)\right]=\left[\frac{k}{g}+\alpha_{1},\frac{l}{g}+\alpha_{2}\right]=\left[\beta_{1},\beta_{2}\right].$$

It is worth highlighting that if $\left(s_{k}=s_{l}\right)$ and $\left(\alpha_{1},\alpha_{2}\right)$, the interval 2-tuple linguistic variable reduces to a 2-tuple linguistic variable.

**Definition** **2.** *Let* $\tilde{a}=\left[\left(r_{1},\alpha_{1}\right),\left(t_{1},\varepsilon_{1}\right)\right]$ *and* $\tilde{b}=\left[\left(r_{2},\alpha_{2}\right),\left(t_{2},\varepsilon_{2}\right)\right]$ *be two interval 2-tuples and* λ∈[0,1]*, then the main operations of interval 2-tuples are expressed as follows [48]:*

*(1)* $\tilde{a}+\tilde{b}=\left[\left(r_{1},\alpha_{1}\right),\left(t_{1},\varepsilon_{1}\right)\right]+\left[\left(r_{2},\alpha_{2}\right),\left(t_{2},\varepsilon_{2}\right)\right]=\Delta \left[\Delta^{-1}\left(r_{1},\alpha_{1}\right)+\Delta^{-1}\left(r_{2},\alpha_{2}\right),\Delta^{-1}\left(t_{1},\varepsilon_{1}\right)+\Delta^{-1}\left(t_{2},\varepsilon_{2}\right)\right];$

*(2)* $\tilde{a}\times \tilde{b}=\left[\left(r_{1},\alpha_{1}\right),\left(t_{1},\varepsilon_{1}\right)\right]\times \left[\left(r_{2},\alpha_{2}\right),\left(t_{2},\varepsilon_{2}\right)\right]\;=\Delta \left[\Delta^{-1}\left(r_{1},\alpha_{1}\right)\cdot \Delta^{-1}\left(r_{2},\alpha_{2}\right),\Delta^{-1}\left(t_{1},\varepsilon_{1}\right)\cdot \Delta^{-1}\left(t_{2},\varepsilon_{2}\right)\right];$

*(3)* $\lambda \tilde{a}=\lambda \left[\left(r_{1},\alpha_{1}\right),\left(t_{1},\varepsilon_{1}\right)\right]=\Delta \left[\lambda \cdot \Delta^{-1}\left(r_{1},\alpha_{1}\right),\lambda \cdot \Delta^{-1}\left(t_{1},\varepsilon_{1}\right)\right].$

**Definition** **3.** *Let* $S=\left\{s_{0},s_{1},...,s_{g}\right\}$ *be a linguistic term set and* $\tilde{a}=\left[\left(s_{k},\alpha_{1}\right),\left(s_{l},\alpha_{2}\right)\right]$ *is an interval 2-tuple, then the interval 2-tuple linguistic variable can be transferred into a crisp value by*

(3) $$\hat{a}=S\left(\tilde{a}\right)=\frac{k+l}{2g}+\frac{\alpha_{1}+\alpha_{2}}{2}$$

*where* $S\left(\tilde{a}\right)\in \left[0,1\right]$ *is the score function of the interval 2-tuple* $\tilde{a}$ *[21].*

### 3.2. Evidential Reasoning Approach

The ER approach is well suited for dealing with multiple criteria decision analysis problems considering the quantitative and qualitative measurements assessed using subjective judgments with uncertainties. Details of the ER approach can be easily found in current literature, see, e.g., [16,17]. Here, we only briefly review some basic concepts which will be used in our proposed decision making approach.

**Definition** **4.** *(Belief Structure). For a frame of discernment* $\hat{H}=\left\{H_{ij},i,j=1,...,n\right\}$*, a belief structure is expressed by*

(4) $$E\left(C\right)=\left\{\left(H_{ij},\beta_{ij}\right),i,j=1,...,n\right\},$$

*where* E(C) *stands for the performance evaluation in terms of a particular criterion, H_ii_ for i = 1,…,n are individual assessment grades defined to classify that criterion, H_ij_ for i = 1,….,n−1 and j = i + 1 to n are the interval assessment grades between H_ii_ and H_jj_, and* $\beta_{ij}$ *are the belief degrees attached to the corresponding assessment grades. A belief degree generally represents the strength to which an answer is believed to be true and it must be equal to or less than 1. If* $\sum_{i,j=1}^{n}\beta_{ij}=1$*, the evaluation is complete, otherwise the evaluation is considered as incomplete.*

**Definition** **5.** *(Rule of Combination). Let* $\left\{\left(H_{ij},\beta_{ij}^{k}\right),i,j=1,...,n;k=1,...,K\right\}$ *be the belief structure of the kth piece of evidence on the frame of discernment* $\hat{H}$*, that has an associated weighing vector* $w={\left(w_{1},w_{2},...,w_{K}\right)}^{T}$*, with* $w_{k}\in \left[0,1\right]$ *and* $\Sigma_{k=1}^{K}w_{k}=1$*, the combination of the K pieces of evidence, called collective belief structure, is denoted as*

(5) $$E_{K}\left(A\right)=\left\{\left(H_{ij},\beta_{ij}^{K}\right),i,j=1,...,n\right\},$$

*where* $\beta_{ij}^{K}$ *is referred to as collective belief degree and determined by*

(6) $$\beta_{ij}^{K}=\sum_{k=1}^{K}w_{k}\beta_{ij}^{k},\;i,j=1,...,n.$$

### 3.3. The DEMATEL Method

The decision making trial and evaluation laboratory (DEMATEL) methodology is an appropriate approach for extracting the interdependent relationships and the intensity of interdependence between complex components of a system [13,14]. The main steps involved in the DEMATEL can be explained as follows:

**Step** **1:** Obtain the initial direct-influence matrix

First, a committee of *l* experts $E=\left\{E_{1},E_{2},...,E_{l}\right\}$ is formed to evaluate the direct influence between different factors based on a numeric scale from 0 to 4, representing “no influence (0)”, “low influence (1)”, “medium influence (2)”, “high influence (3)”, and “very high influence (4)”, respectively. As the result of evaluation, the initial direct-influence matrix $Z_{k}={\left[z_{ij}^{k}\right]}_{n\times n}$ can be obtained for each expert, where all principal diagonal elements are equal to zero and $z_{ij}^{k}$ represents the judgment provided by the *k*th expert on the degree to which factor *i* affects factor *j*. By aggregating all the experts’ opinions, the group direct-influence matrix $Z={\left[z_{ij}\right]}_{n\times n}$ is computed by

(7) $$z_{ij}=\frac{1}{l}\sum_{k=1}^{l}z_{ij}^{k},\;i,j=1,2,...,n.$$

**Step** **2:** Calculate the normalized direct-influence matrix

The normalized direct-influence matrix $X={\left[x_{ij}\right]}_{n\times n}$ is calculated through Equations (8)–(9).

(8) $$X=\frac{Z}{s},$$

(9) $$s=\max\left\{\underset{1\leq i\leq n}{\max}\sum_{j=1}^{n}z_{ij},\underset{1\leq j\leq n}{\max}\sum_{i=1}^{n}z_{ij}\right\},$$

where *s* denotes the biggest value among the sums of each row and each column.

**Step** **3:** Derive the total-influence matrix

Once the normalized direct-influence matrix *X* is obtained, the total-influence matrix $T={\left[t_{ij}\right]}_{n\times n}$ can be derived by summing all direct and indirect effects as shown in Equation (10).

(10) $$T\;=\underset{k\to \infty }{\lim}\left(X+X^{2}+\cdots +X^{h}\right)=X{\left(I-X\right)}^{-1},$$

where *I* is the identity matrix.

**Step** **4:** Build the influential relation diagram (IRD)

Using the values of *R* + *C* and *R* – *C*, the IRD can be mapped, where *R* and *C* are the sum of the rows and the sum of the columns in the total-influence matrix *T*, respectively.

(11) $$R={\left[r_{i}\right]}_{n\times 1}={\left[\sum_{j=1}^{n}t_{ij}\right]}_{n\times 1},$$

(12) $$C={\left[c_{j}\right]}_{n\times 1}={\left[\sum_{i=1}^{n}t_{ij}\right]}_{1\times n}^{T}.$$

Note that *r_i_* denotes the sum of all direct and indirect influences dispatched from factor *i* to other factors and is called the degree of influential impact. In contrast, *c_j_* represents both direct and indirect impacts that factor *j* receives and is called the degree of influenced impact. 

Let *i* = *j* and i,j∈{1,2,...,n}; the value of *r_i_* + *c_j_* named “prominence” indicates the total effects given and received by the *i*th factor. That is, it represents both the *i*th factor’s impact on the whole system and other factors’ impact on the *i*th factor. So, *r_i_* + *c_j_* is a measure of importance degree of the *i*th factor in the entire system. Similarly, the difference *r_i_* − *c_j_* called “relation” shows the net effect that contributed by the *i*th factor to the system. Specifically, if *r_i_* − *c_j_* > 0, the *i*th factor is a net cause and belongs to the cause group, and if *r_i_* − *c_j_* < 0, the *i*th factor is a net receiver and belongs to the effect group. Finally, an IRD can be plotted using *r_i_* + *c_j_* as the horizontal axis and *r_i_* − *c_j_* as the vertical axis. Moreover, the entries in the matrix *T* can be mapped into the diagram by using solid lines and broken lines for two-way and one-way significant relationships, respectively. 

Above, an IRD is constructed based on the matrix *T* to explain the structure relations of factors. However, in some situations, the IRD may be too complex to show valuable information for decision making if all the relations are considered. Therefore, a threshold value *θ* is often set to filter out negligible effects to diminish this complexity of the whole system. That is, only the elements of the matrix *T*, whose influence level is greater than the threshold value, are selected and converted into the IRD. If the threshold value is too low, many factors are included and the IRM will be too complex to comprehend. On the contrary, some important factors may be excluded if the threshold value is too high. In this study, the threshold value is determined by computing the average of the matrix *T* [12].

## 4. The Proposed Linguistic Evidential DEMATEL Approach

**Figure 1:**
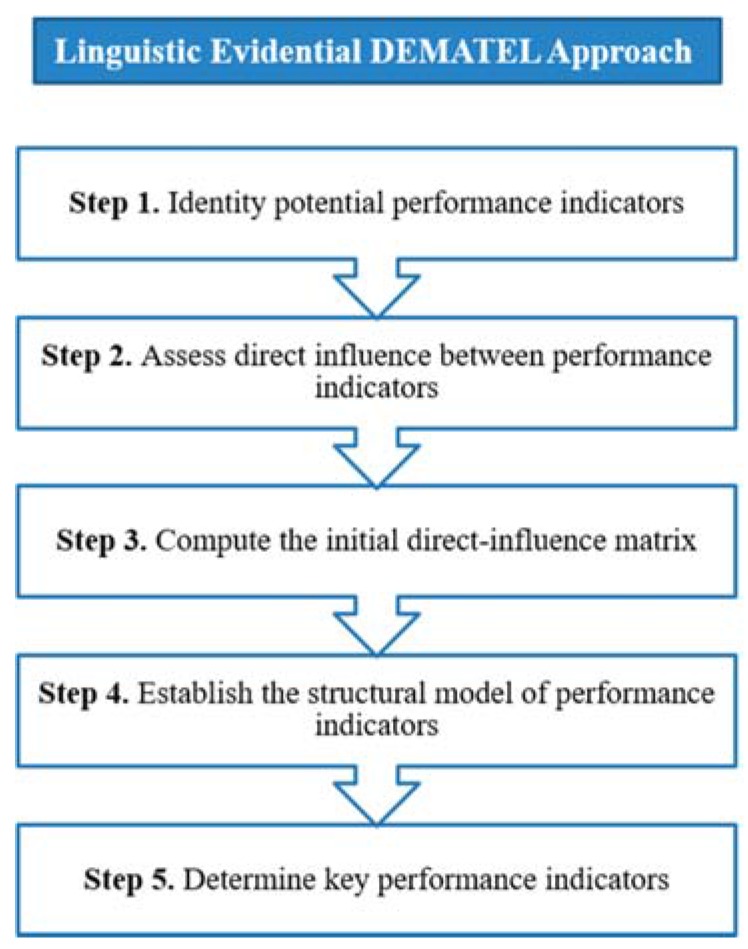
Framework of the linguistic evidential DEMATEL approach.

Quantifying the interrelationships of indicators and identifying key performance indicators (KPIs) for healthcare management can be considered as a group decision behavior. In many complex systems, subjective evaluations of experts are always expressed in linguistic terms rather than crisp values. Furthermore, considering their different expertise and backgrounds, decision makers may provide different types of assessment information for the interdependent relationships between performance indicators, some of which may be precise or imprecise, certain or uncertain, and complete or incomplete. Under such circumstances, the classical decision making trial and evaluation laboratory (DEMATEL) method cannot be applied directly to figure out the KPIs in relation to the success of healthcare performance measurement. In this section, we propose a linguistic evidential DEMATEL approach to address the uncertainties of human judgments to identify the importance and classification of performance indicators. The flowchart in Figure 1 shows the general framework of the proposed approach. In this framework, belief structures of the evidential reasoning (ER) approach are implemented to express ambiguous concepts associated with decision makers’ subjective judgments. Then, the interval 2-tuple linguistic model is utilized to aggregate individual evaluations of direct influence of performance indicators into group opinions. At last, the DEMATEL is applied on the acquired results to identify a minimum set of KPIs for holistic hospital management.

The procedure of the proposed linguistic evidential DEMATEL is summarized as follows:

**Step** **1.** Determine possible performance indicators influencing system objective

A systematic review of literature is required to search and collect relevant information at this stage. Additionally, it is necessary to establish a committee of domain experts, who provide group knowledge for related issues. In this step, possible performance indicators used for measuring and managing healthcare systems should be determined based on collected information and experts’ opinions.

**Step** **2.** Invite experts to assess direct influence between performance indicators

After the agreement about initial performance indicators is formed, questionnaire survey of the healthcare experts should be conducted to investigate the interaction between each pair of indicators. As a result, the linguistic assessments regarding the direct influence which indicators have impacts on each other can be obtained.

As mentioned before, different types of uncertainties are inevitably involved in the judgments the expert group provided on the interrelationships among indicators due to the subjectivity and incompleteness. Such a problem can be solved using the ER approach, which can capture information of different forms and accommodate uncertainties of different types under a unified framework. Suppose the individual evaluation grade set *H* is defined as

(13) $$H=\left\{H_{11},H_{22},H_{33},H_{44},H_{55}\right\}=\left\{Very\;Low,Low,Moderate,High,Very\;High\right\},$$

then experts can provide their subjective judgments in the following flexible ways:

- A certain grade such as *Low*, which can be expressed as $\left\{\left(H_{22},1.0\right)\right\}$.
- A distribution such as *Low* to a degree of belief 0.3 and *Moderate* to a degree of belief 0.7 can be equivalently written as $\left\{\left(H_{22},0.3\right),\left(H_{33},0.7\right)\right\}$. Here the values of 0.3 and 0.7 represent the belief degrees (also called confidences) of the expert in his/her subjective judgments.
- An interval such as *Low*-*Moderate*, which means that the evaluation grade given by the expert is between *Low* and *Moderate*. This can be expressed as $\left\{\left(H_{23},1.0\right)\right\}$
- No judgment, which means the expert is not willing to or cannot provide an assessment due to a lack of evidence or data. In other words, the grade by this expert could be anywhere between *Very Low* and *Very High* and can be represented as $\left\{\left(H_{15},1.0\right)\right\}$.

**Step** **3.** Aggregate experts’ assessments to compute the initial direct-influence matrix

Aggregate the linguistic evaluations of experts into group assessments by applying the combination rule of Equations (5)–(6) to every element of the linguistic belief decision matrixes. Then convert the obtained collective belief structures into interval 2-tuples using the weighted sum method and turn them into crisp scores by Equation (3). In this way, we can obtain $z_{ij}$, which represents the direct impact of indicator *i* on indicator *j*. Thereby, an initial direct-influence matrix $Z={\left[z_{ij}\right]}_{n\times n}$ can be constructed, where *Z* is a nonnegative matrix.

**Step** **4.** Draw IRD and establish the structural model of performance indicators

Based on the initial direct-influence matrix *Z*, the total-influence matrix *T* can be easily calculated by applying the DEMATEL approach in Equations (8)–(10). Then the importance degree and net effect degree are computed for each performance indicator to construct the IRD; thus the structural relationship and importance of performance indicators can be visualized.

**Step** **5.** Analyze the structure of performance indicators to identify KPIs

Analyze each performance indicator in comprehensive consideration of the indexes *r_i_*, *c_i_*, *r_i_* + *_i_*, *r_i_* − *c_i_* and the visualized IRD. According to the position of each performance indicator in the whole system, the ones which have great effect on other indicators or have complicated relationship with other indicators can be identified. Clearly, performance indicators of this kind are KPIs and improvement of them would bring improvement to the overall healthcare system.

## 5. Case Study

### 5.1. Application

Based on a systematic literature review on healthcare performance assessment, the performance indicators frequently used in previous studies were identified from the perspectives of patient, employee and management [1]. The literature survey searched PubMed and PubMed Central databases for articles applying performance indicators for the measurement of healthcare performance. As a result, 24 relevant papers were elicited according to the predefined screening criteria, from which a total of 428 indicators were extracted. Among them, 15 indicators appeared in five or more of the selected studies are considered as “frequently used” indicators (cf. Table 1). Next, these performance indicators are analyzed and structured following the procedure of our proposed linguistic evidential decision making trial and evaluation laboratory (DEMATEL) approach.

To measure interrelationships among the 15 performance indicators, surveys and interviews were conducted in a university hospital to make assessments in terms of influence and relationship. For doing so, an expert committee of five decision makers was built to complete the questionnaire. These experts from different departments or institutions included a head nurse and a medical doctor who is responsible for an internal medicine department, a hospital leader and a deputy hospital leader who are also physicians, and a professor of industrial engineering who is researcher on healthcare operation management. All these decision makers possess professional knowledge of healthcare performance measurement and have worked in related fields for more than three years. To reflect their differences in decision-making, the five experts were assigned the following weights: 0.15, 0.20, 0.25, 0.25 and 0.15, respectively. In addition, due to the difficulty in precisely assessing the interdependency of indicators, the experts agreed to evaluate them using the linguistic term set *S*:

(14) $$\begin{array}{l} S=\{s_{0}=No\;influence,s_{1}=Very\;low\;influence,s_{2}=Low\;influence,s_{3}=Medium\;influence,\\ \;s_{4}=High\;influence,s_{5}=Very\;high\;influence,s_{6}=Extremely\;high\;influence\}. \end{array}$$

The five decision makers gave their linguistic assessments about the direct influence between each pair of performance indicators. Because it was different from one to another and included incomplete information, the experts’ assessments were expressed by using linguistic belief structures. For example, the initial direct-influence matrix given by the first expert $Z^{1}$ is presented in Table 2. Next, the linguistic belief structures are aggregated into collective belief structures using Equations (5) and (6). Table 3 displays the group assessments of the five experts on the patient related indicators. Then Equation (3) is applied to transform the interval 2-tuple linguistic matrix into crisp values to construct the initial direct-relation matrix $Z={\left[z_{ij}\right]}_{15\times 15}$ as in Table 4. 

**Figure 2:**
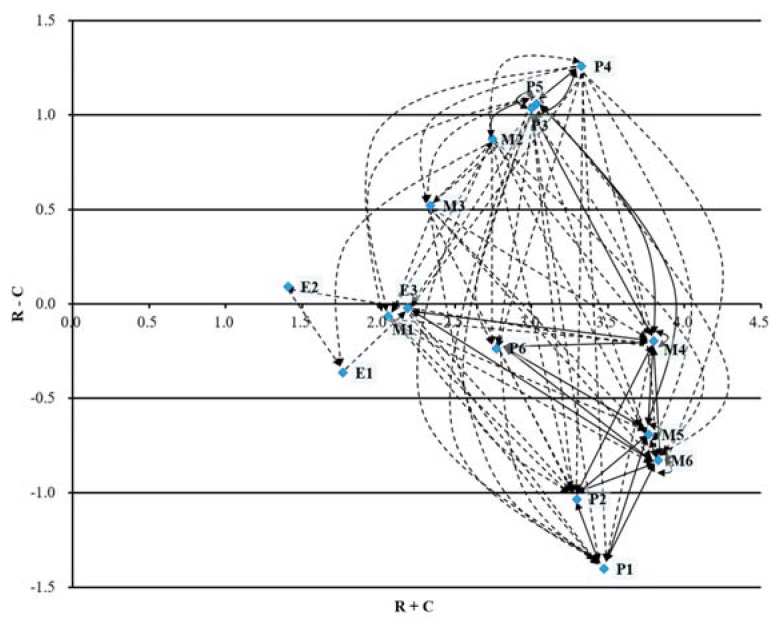
Influential relation diagram for the example.

Next, the data of the interrelationships between performance indicators are were analyzed with the DEMATEL approach. The total-influence matrix *T* is computed by Equations (8)–(10) and given in Table 5. Then, the indexes of *R* + *C* and *R* – *C* of each performance indicator are computed via Equations (11)–(12). The computed results are summarized in Table 6. Finally, an IRD of the 15 indicators is acquired as shown in Figure 2. Note that the arithmetic mean of the matrix *T* (i.e., 0.0951) is used as threshold value to filter out weaker influential relations among the performance indicators. 

As seen in Figure 2, the 15 indicators are visually divided into two groups according to whether their values of *r_i_* − *c_i_* are positive or negative. As a result, the cause group with positive *r_i_* − *c_i_* values includes P4, P5, P3, M2, M3, E2, and other indicators including E3, M1, M4, P6, E1, M5, M6, P2, P1 are in the effect group. There are many other valuable clues that can be acquired from Figure 2 to facilitate making healthcare improvement decisions. In the following, the IRD will be discussed in detail, and the KPIs for holistic healthcare management are identified taking into account the scores in Table 6.

### 5.2. Discussions

Among the indicators of the cause group, “accidents/adverse events” (P4) has the highest value of *r_i_* − *c_i_*, which implies that P4 exerts more effect on the whole system than that it receives from other indicators. In addition, as shown in Table 6, the degree of influential impact of P4 is 2.293, which ranks first among all the causal indicators. It means that P4 has great effect on other indicators and that improvement of P4 can lead to improvement of the overall system. To enhance the performance of healthcare system, a low rate of accidents/adverse events should be first achieved. Therefore, P4 is a KPI that should be paid more attention to in the healthcare performance measurement system. Meanwhile, “nosocomial infection” (P5) and “incidents/errors” (P3) can be clustered as KPIs for similar reasons. 

With respect to “number of operations/procedures” (M2), its net effect value *r_i_* − *c_i_* is in the fourth place among the cause indicators and its influential impact index *r_i_* is relatively high. The impact given by M2 to the whole system is great, and optimization of M2 will definitely improve the effectiveness and efficiency of the healthcare system. Hence, it is reasonable to identify “number of operations/procedures” (M2) as one of the KPIs. 

Next, the characteristics of effect indicators are analyzed to figure out possible KPIs. Among the performance indicators, “financial measures” (M6) has the highest value of *r_i_* + *c_i_*, indicating that it is quite important for the healthcare management system. However, as can be seen in Table 6, the *r_i_* − *c_i_* value of M6 is −0.829, a value less than zero showing M6 is a net effect indicator. To further elucidate this scenario, its *r_i_* and *c_i_* values (i.e., 1.499 and 2.328) are relatively high in all the indicators. This denotes that although M6 is a net receiver, it plays a significant role in promoting and improving healthcare management activities. Thus, “financial measures” (M6) is a KPI considering its complex relationships with other indicators. Following the similar logic, “length of stay” (M4) and “bed occupancy” (M5) can be considered as KPIs in the healthcare system.

Based on the aforementioned discussions, seven KPIs for hospital performance management from the patient, employment and management perspectives are identified. These KPIs include four–cause indicators and three–effect indicators which are “accidents/adverse events”, “nosocomial infection”, “incidents/errors”, “number of operations/ procedures”, “length of stay”, “bed occupancy”, and “financial measures”. Note that depending on the particular hospital applied, the assessments of performance indicators may be dissimilar and thus different KPIs may be derived. However, as for the considered case study, it is a favorable way to improve the healthcare performance through optimizing the seven KPIs. Additionally, the identified KPIs can be considered as crucial criteria to improve the efficiency of healthcare management during the planning phase.

To verify the performance of the proposed hybrid MCDM approach, we gathered medical doctors and managers to check the obtained KPIs. According to the domain experts, the proposed linguistic evidential DEMATEL model is highly suitable for the considered application to analyze the cause-effect relationships among indicators and identify KPIs which are of more fundamental importance for the whole healthcare system. Accordingly, by monitoring and managing these KPIs, the effects of prevention, continual improvements, and innovations can be achieved to shape core competitiveness of the healthcare organization.

## 6. Conclusions

In this paper we introduced a novel performance indicator identification and assessment framework, named as linguistic evidential decision making trial and evaluation laboratory (DEMATEL), for holistic hospital management. This model extends the traditional DEMATEL method to capture decision information of different forms and to accommodate uncertainties of different types by applying the evidential reasoning (ER) approach and interval 2-tuple linguistic variables. It successfully determined the importance of each indicator and acquired the causal relationships between performance indicators. As a result, a set of complex indicators were separated into cause and effect groups and a visible influential relation diagram (IRD) was formed. Based on the results of the case study, seven key performance indicators (KPIs) were defined for the healthcare performance management. Therefore, hospital managers can apply a phased quality control method to improve the healthcare performance gradually with the limited resources. 

Despite its contributions, this study has several limitations, which may be addressed by future research. First, the weights of domain experts in the proposed approach are determined through a direct assignment method. In many practical situations, however, the information of expert weights is completely unknown or partially known. Therefore, future research should be conducted in developing an optimal model to derive expert weights objectively. Second, in the case study, the data are collected from a small number of healthcare experts, which may limit the generalization of its findings to other hospitals. Thus, in the future, more case studies with accepted number of participants can be carried out to improve or further validate the findings of this study. Additionally, the proposed model of identifying KPIs for healthcare management can be easily applied to diverse real applications, such as determining critical success factors of emergency management and analyzing major internal barriers in electric vehicle industrialization. 

## Figures and Tables

**Table 1:** Performance indicators frequently used in previous studies.

Perspective	Measure	Indicator	Definition and Explanation
Patient	Patient satisfaction	Overall satisfaction (P1)	Overall satisfaction with healthcare service, including satisfaction with physicians, waiting time, treatment, etc.
	Patient complaints	Overall complaints (P2)	Overall complaints about healthcare service, e.g., patient complaints per 1000 patient, rate of complaints per patient per year
	Patient safety	Incidents/Errors (P3)	Incidents/errors occurred in healthcare treatment process, including medication errors, diagnosis and treatment errors, blood transfusion errors, etc.
		Accidents/adverse events (P4)	Accident/adverse events occurred in healthcare treatment process, e.g., number of critical incidents per 100 operations
		Nosocomial infection (P5)	Nosocomial infection in the hospital, including surgical wound infection, infection of Methicillin-resistant Staphylococcus aureus (MRSA), incision-wound infection, etc.
	Waiting/Delay	Waiting time (P6)	Waiting time for healthcare service, such as outpatient waiting times, waiting time for admission, waiting time for treatment
Employee	Employee satisfaction	Overall satisfaction (E1)	Overall satisfaction with healthcare organization, including satisfaction with job, colleagues, supervisors etc.
	Occupational health	Sickness leave (E2)	Employee sick leave in the hospital, such as sickness leave of doctor, mental sickness, physical sickness
	Work conditions	Staff turnover (E3)	Staff turnover of the hospital, e.g., staff leaving the hospital in one year/total staffs
Management	Health statistics	Mortality/Death (M1)	Mortality/Death in healthcare organization, such as mortality of the patients discharged, stillbirths and infant deaths, deaths in hospital following surgery
		Number of operations/ procedures (M2)	Number of operations/procedures carried out in the hospital per operator/year
	Readmission/return	Unscheduled readmission/return (M3)	Unscheduled readmission/return to the hospital, e.g., readmissions within 28 days, readmission rate within 14 days, unexpected returns after transferred out
	Organizational efficiency	Length of stay (M4)	Length of stay at the hospital, e.g., average length of stay, rate of length of stay exceeding 14 days, per cent long stay patient
		Bed occupancy (M5)	Bed occupancy of the hospital, such as average daily census/beds in service, percentage of bed emptiness
	Financial effectiveness	Financial measures (M6)	Financial measures for healthcare organisation, including cash-flow, profit margin, net operating margin, asset turnover, return on assets, etc.

**Table 2:** Initial direct-influence matrix of the first expert.

DM1	P1	P2	P3	P4	P5	P6	E1	E2	E3	M1	M2	M3	M4	M5	M6
P1	NA	H_55_	H_22_	H_12_	H_22_	H_23_	(H_44_, 0.7) (H_55_, 0.3)	H_22_	H_22_	H_22_	H_12_	H_11_	H_22_	H_66_	H_56_
P2	H_67_	NA	H_22_	(H_11_, 0.5)(H_22_, 0.3)	H_11_	H_33_	H_22_	H_11_	H_12_	H_12_	H_33_	H_22_	H_44_	H_55_	(H_55_,0.6) (H_66_,0.4)
P3	H_66_	H_66_	NA	H_55_	H_44_	H_44_	H_33_	H_33_	H_22_	H_55_	H_22_	H_55_	H_56_	H_44_	H_55_
P4	H_77_	H_77_	H_55_	NA	H_44_	H_55_	H_33_	H_44_	H_33_	H_66_	H_22_	H_55_	H_56_	H_55_	H_56_
P5	H_66_	H_45_	H_55_	H_55_	NA	H_33_	H_22_	H_11_	H_11_	H_66_	H_44_	H_55_	H_55_	H_55_	H_44_
P6	(H_44_, 0.5) (H_55_, 0.5)	H_55_	H_12_	H_11_	H_33_	NA	H_22_	H_11_	H_11_	H_12_	H_44_	H_11_	H_55_	H_55_	H_45_
E1	H_33_	H_33_	H_22_	H_22_	H_11_	H_22_	NA	H_22_	H_66_	H_12_	H_11_	H_11_	H_22_	H_11_	H_22_
E2	H_22_	H_11_	H_22_	H_22_	H_11_	H_34_	(H_55_, 0.4) (H_66_, 0.6)	NA	H_55_	H_11_	H_22_	H_11_	H_44_	H_11_	H_22_
E3	H_33_	H_33_	H_44_	H_33_	H_33_	H_33_	H_44_	H_22_	NA	H_22_	H_23_	H_11_	H_33_	H_22_	H_33_
M1	H_55_	H_44_	H_22_	H_22_	H_11_	H_11_	H_11_	H_11_	H_11_	NA	H_12_	H_22_	H_34_	H_55_	H_55_
M2	H_33_	H_33_	H_33_	H_33_	H_33_	H_55_	H_44_	H_22_	H_22_	H_34_	NA	H_44_	(H_44_, 0.8)	H_55_	H_66_
M3	H_66_	H_55_	H_22_	H_22_	H_22_	H_11_	H_11_	H_11_	H_22_	H_55_	H_45_	NA	H_55_	H_44_	H_44_
M4	H_55_	H_55_	H_34_	H_33_	H_55_	H_55_	H_22_	H_45_	H_44_	H_33_	H_22_	H_23_	NA	H_55_	H_55_
M5	H_44_	H_44_	H_33_	H_22_	H_45_	H_55_	H_11_	H_33_	H_44_	H_22_	H_22_	H_22_	H_56_	NA	H_55_
M6	H_66_	H_55_	H_22_	H_22_	H_22_	H_44_	H_44_	H_11_	H_44_	H_22_	H_11_	H_11_	H_44_	H_55_	NA

DM: decision Maker; NA: Not Applicable.

**Table 3:** Group assessment of the experts on patient related indicators.

Indicators	P1	P2	P3	P4	P5	P6
P1	(H_11_, 1.0)	(H_44_, 0.6) (H_55_, 0.4)	(H_11_, 0.4) (H_22_ 0.6)	(H_11_, 0.35) (H_12_, 0.40) (H_22_, 0.25)	(H_11_, 0.40) (H_12_, 0.20) (H_22_, 0.40)	(H_22_, 0.48) (H_23_, 0.15) (H_33_, 0.37)
P2	(H_66_, 0.7) (H_67_, 0.3)	(H_11_, 1.0)	(H_11_, 0.4) (H_22_, 0.6)	(H_11_, 0.325) (H_17_, 0.055) (H_22_, 0.62)	(H_11_, 1.0)	(H_22_, 0.65) (H_23_, 0.20) (H_33_, 0.15)
P3	(H_55_, 0.25) (H_66_, 0.75)	(H_55_, 0.4) (H_66_, 0.6)	(H_11_, 1.0)	(H_44_, 0.5) (H_55_, 0.5)	(H_33_, 0.50) (H_44_, 0.30) (H_55_, 0.20)	(H_33_, 0.65) (H_44_, 0.35)
P4	(H_66_, 0.25) (H_77_, 0.75)	(H_66_, 0.25) (H_77_, 0.75)	(H_44_, 0.40) (H_45_, 0.20) (H_55_, 0.40)	(H_11_, 1.0)	(H_33_, 0.25) (H_44_, 0.50) (H_55_, 0.25)	(H_44_, 0.85) (H_55_, 0.15)
P5	(H_55_, 0.65) (H_66_, 0.35)	(H_44_,0.50) (H_45_,0.30) (H_55_, 0.20)	(H_44_, 0.40) (H_55_, 0.60)	(H_55_, 1.0)	(H_11_, 1.0)	(H_33_, 0.55) (H_34_, 0.20) (H_44_, 0.25)
P6	(H_44_, 0.325) (H_55_, 0.675)	(H_55_, 0.6) (H_66_, 0.4)	(H_11_, 0.40) (H_12_, 0.40) (H_22_, 0.20)	(H_11_, 1.0)	(H_33_, 0.75) (H_44_, 0.25)	(H_11_, 1.0)

**Table 4:** Initial direct-influence matrix *Z*.

Indicators	P1	P2	P3	P4	P5	P6	E1	E2	E3	M1	M2	M3	M4	M5	M6
P1	0.000	0.567	0.100	0.075	0.083	0.241	0.491	0.067	0.125	0.158	0.071	0.067	0.108	0.733	0.783
P2	0.858	0.000	0.100	0.131	0.000	0.208	0.192	0.000	0.054	0.046	0.258	0.108	0.413	0.750	0.735
P3	0.792	0.767	0.000	0.583	0.450	0.392	0.213	0.225	0.167	0.567	0.213	0.592	0.746	0.500	0.667
P4	0.958	0.958	0.583	0.000	0.500	0.525	0.250	0.292	0.233	0.733	0.167	0.675	0.725	0.667	0.721
P5	0.725	0.558	0.600	0.667	0.000	0.392	0.092	0.042	0.000	0.750	0.425	0.667	0.667	0.667	0.517
P6	0.613	0.733	0.067	0.000	0.375	0.000	0.167	0.000	0.000	0.092	0.425	0.000	0.667	0.525	0.592
E1	0.233	0.233	0.167	0.167	0.075	0.175	0.000	0.167	0.750	0.033	0.000	0.067	0.167	0.075	0.167
E2	0.167	0.075	0.092	0.058	0.042	0.367	0.694	0.000	0.558	0.000	0.058	0.000	0.417	0.075	0.167
E3	0.217	0.258	0.400	0.333	0.225	0.250	0.500	0.167	0.000	0.067	0.213	0.042	0.367	0.233	0.333
M1	0.667	0.575	0.067	0.167	0.000	0.000	0.100	0.000	0.067	0.000	0.033	0.167	0.367	0.625	0.608
M2	0.333	0.367	0.375	0.400	0.450	0.600	0.500	0.233	0.108	0.358	0.000	0.383	0.542	0.667	0.800
M3	0.727	0.550	0.167	0.167	0.067	0.200	0.000	0.000	0.125	0.583	0.567	0.000	0.667	0.529	0.500
M4	0.667	0.546	0.433	0.267	0.450	0.667	0.067	0.554	0.542	0.250	0.250	0.233	0.000	0.667	0.667
M5	0.558	0.500	0.258	0.167	0.533	0.608	0.000	0.333	0.400	0.075	0.233	0.192	0.733	0.000	0.583
M6	0.767	0.667	0.083	0.450	0.083	0.400	0.425	0.067	0.533	0.233	0.250	0.133	0.500	0.667	0.000

**Table 5:** Total-influence matrix *T*.

Indicators	P1	P2	P3	P4	P5	P6	E1	E2	E3	M1	M2	M3	M4	M5	M6
P1	0.072	0.127	0.037	0.038	0.038	0.072	0.087	0.027	0.052	0.044	0.035	0.031	0.071	0.148	0.156
P2	0.175	0.071	0.041	0.047	0.033	0.076	0.057	0.023	0.045	0.035	0.059	0.038	0.109	0.160	0.161
P3	0.234	0.214	0.055	0.124	0.107	0.131	0.083	0.064	0.081	0.126	0.079	0.119	0.197	0.191	0.213
P4	0.271	0.251	0.127	0.065	0.119	0.156	0.094	0.075	0.096	0.151	0.082	0.134	0.210	0.226	0.237
P5	0.231	0.196	0.125	0.136	0.059	0.133	0.067	0.044	0.061	0.151	0.105	0.132	0.193	0.213	0.201
P6	0.159	0.161	0.043	0.038	0.079	0.057	0.057	0.025	0.041	0.046	0.083	0.031	0.145	0.146	0.155
E1	0.073	0.068	0.040	0.040	0.028	0.049	0.024	0.033	0.110	0.023	0.018	0.024	0.057	0.051	0.064
E2	0.065	0.051	0.032	0.028	0.026	0.074	0.105	0.016	0.094	0.018	0.025	0.015	0.086	0.051	0.064
E3	0.100	0.097	0.077	0.072	0.058	0.077	0.091	0.041	0.037	0.041	0.053	0.034	0.103	0.095	0.109
M1	0.147	0.128	0.033	0.047	0.027	0.044	0.041	0.019	0.041	0.026	0.030	0.042	0.096	0.138	0.138
M2	0.166	0.156	0.093	0.099	0.104	0.147	0.109	0.062	0.072	0.096	0.049	0.091	0.165	0.191	0.210
M3	0.183	0.152	0.057	0.060	0.049	0.086	0.043	0.028	0.058	0.107	0.103	0.034	0.154	0.157	0.157
M4	0.199	0.173	0.098	0.083	0.102	0.153	0.065	0.097	0.117	0.080	0.078	0.071	0.102	0.190	0.194
M5	0.171	0.152	0.073	0.065	0.105	0.137	0.048	0.068	0.093	0.054	0.070	0.061	0.170	0.100	0.169
M6	0.189	0.167	0.051	0.092	0.053	0.110	0.094	0.037	0.109	0.067	0.067	0.051	0.138	0.171	0.101

**Table 6:** Sum of influences given and received for each performance indicator.

Performance indicator	R	C	R + C	R – C
P1. Patient satisfaction	1.035	2.438	3.472	−1.403
P2. Patient complaints	1.131	2.165	3.296	−1.034
P3. Incidents/Errors	2.019	0.983	3.002	1.036
P4. Accidents/adverse events	2.293	1.035	3.328	1.258
P5. Nosocomial infection	2.047	0.988	3.035	1.059
P6. Waiting time	1.267	1.503	2.770	−0.236
E1. Employee satisfaction	0.701	1.065	1.766	−0.364
E2. Sickness leave	0.750	0.659	1.409	0.091
E3. Staff turnover	1.084	1.106	2.190	−0.022
M1. Mortality/Death	0.999	1.064	2.063	−0.065
M2. Number of operations/procedures	1.809	0.937	2.746	0.872
M3. Unscheduled readmission/return	1.430	0.908	2.337	0.522
M4. Length of stay	1.802	1.998	3.800	−0.195
M5. Bed occupancy	1.538	2.229	3.767	−0.691
M6. Financial measures	1.499	2.328	3.827	−0.829

R: The sum of the rows in the matrix *T* ; C: The sum of the columns in the matrix *T* .
